# Detection of large rearrangements in a hereditary pan-cancer panel using next-generation sequencing

**DOI:** 10.1186/s12920-019-0587-3

**Published:** 2019-10-17

**Authors:** Debora Mancini-DiNardo, Thaddeus Judkins, John Kidd, Ryan Bernhisel, Courtney Daniels, Krystal Brown, Kirsten Meek, Jonathan Craft, Jayson Holladay, Brian Morris, Benjamin B. Roa

**Affiliations:** Myriad Genetic Laboratories, Inc, 320 Wakara Way, Salt Lake City, UT 84108 USA

**Keywords:** Large rearrangements, Next-generation sequencing, Hereditary pan-cancer panel testing, Confirmatory testing, Deletion, Duplication, Microarray-CGH, MLPA

## Abstract

**Background:**

Healthcare providers increasingly use information about pathogenic variants in cancer predisposition genes, including sequence variants and large rearrangements (LRs), in medical management decisions. While sequence variant detection is typically robust, LRs can be difficult to detect and characterize and may be underreported as a cause for hereditary cancer risk. This report describes the outcomes of hereditary cancer genetic testing using a comprehensive strategy that employs next-generation sequencing (NGS) for LR detection, coupled with LR confirmation using repeat hybrid capture NGS, microarray comparative genomic hybridization (microarray-CGH), and/or multiplex ligation-dependent probe amplification (MLPA).

**Methods:**

Sequencing and LR analysis were conducted in a consecutive series of 376,159 individuals who received clinical testing with a hereditary pan-cancer gene panel from September 2013 through May 2017. NGS dosage analysis was used to evaluate potential deletions or duplications, with controls in place to exclude pseudogene reads. Samples positive for a putative LR based on NGS were confirmed using a comprehensive approach that included targeted microarray-CGH and/or MLPA analysis, with further examination as needed to ascertain the nature of the LR.

**Results:**

A total of 3461 LRs were identified and classified as a deleterious mutation (DM), suspected deleterious mutation (SDM) or variant of uncertain significance. Pathogenic LRs (DM/SDM) accounted for the majority of LRs (67.7%), the largest proportion of which were deletions (86.1%), followed by duplications (11.3%), insertions (1.8%), triplications (0.5%), and inversions (0.3%). Several cases presented illustrate that the laboratory approach employed here can ensure consistent identification and accurate characterization of LRs. In the absence of this comprehensive testing strategy, 9% of LRs identified in this testing population might have been missed, potentially leading to inappropriate medical management in as many as 210 individuals referred for hereditary cancer testing.

**Conclusions:**

These data show that copy number analysis using NGS coupled with confirmatory testing reliably detects and characterizes LRs. Further, LRs comprise a substantial proportion (7.2%) of pathogenic variants identified by the test. A robust and accurate LR identification strategy is an essential component of a high-quality genetic testing program, enabling clinicians to optimize patient medical management decisions.

## Background

Large rearrangements (LRs) are a class of genetic variations that include deletions, duplications, triplications, and retroelement insertions. LRs are considered pathogenic when they disrupt critical regions of a gene and impair its function. Along with sequence mutations, pathogenic LRs in genes associated with hereditary cancer syndromes correlate with increased cancer risk. For example, pathogenic *BRCA1* and *BRCA2* variants are associated with a lifetime breast cancer risk of 43–87% and a lifetime ovarian cancer risk of 16–63% [1–5]. While sequence variant detection typically is comprehensive, LRs can be difficult to detect and characterize. As a result, LRs may be an underreported and underappreciated cause for hereditary cancer risk.

Large deletions and duplications have traditionally been detected using technologies such as multiplex ligation-dependent probe amplification (MLPA) and microarray-based comparative genomic hybridization (microarray-CGH), both of which have advantages and disadvantages [6, 7]. MLPA is limited in its ability to detect certain LRs due to the number and placement of probes (i.e., incomplete or indirect exonic coverage) [6]. While microarray-CGH detects most unbalanced LRs adeptly, particularly partial deletions and duplications, it cannot directly detect retroelement insertions (e.g. Alu insertions) [8].

Next-generation sequencing (NGS) has enabled the simultaneous identification of sequence variants, as well as copy number analysis changes across multiple genes. Our laboratory-developed NGS panel was validated for accurate sequence analysis as compared with Sanger sequencing, along with accurate LR detection [9]. The comprehensive LR analysis strategy involves NGS dosage analysis as a robust first-line test, followed by confirmation via repeat hybrid capture NGS, microarray-CGH, MLPA, and/or long-range PCR analyses [9]. While straightforward deletions and duplications can be simply confirmed through repeat hybrid capture NGS dosage analysis, LRs that are more difficult to characterize benefit from this combined approach, which complements the strengths and compensates for the limitations of any one detection method. For instance, any putative LR detected in exons 12–15 in *PMS2* requires additional analyses to ensure that only clinically significant PVs are reported. Therefore, the availability of multiple detection methods is desirable for comprehensive and accurate characterization of LRs.

This analysis paired a comprehensive LR detection strategy with an NGS-based, clinically-validated hereditary pan-cancer panel test. The frequency and distribution of LRs identified across all panel genes are reported for a large clinical testing cohort.

## Methods

This analysis examined a consecutive series of 376,159 individuals who received clinical testing with a hereditary pan-cancer panel test at our Clinical Laboratory Improvement Amendments and College of American Pathology approved laboratory (Myriad Genetic Laboratories, Inc.) from September 2013 through May 2017. All individuals provided written informed consent for clinical testing, and the data presented here were de-identified for analysis. No additional information was obtained from patients or healthcare providers for this analysis.

### Genetic testing

The hereditary pan-cancer panel test used in this analysis has been analytically validated and described in detail previously [9]. Panel genes included *APC, ATM, BARD1, BMPR1A, BRCA1, BRCA2, BRIP1, CDH1, CDK4, CDKN2A* (p16INK4a and p14ARF)*, CHEK2, EPCAM, GREM1, MLH1, MSH2, MSH6, MUTYH, NBN, PALB2, PMS2, POLD1*, *POLE, PTEN, RAD51C*, *RAD51D*, *SMAD4, STK11,* and *TP53.* Testing included sequencing and LR analysis of all genes except *POLD1* and *POLE*, for which only sequencing of the exonuclease domains was performed. For *EPCAM* and *GREM1*, only LR analysis was performed. The panel became available in September 2013 and included all listed genes except *POLD1*, *POLE*, and *GREM1*, which were added in July 2016. For testing, germline DNA from blood or saliva samples were processed through a PCR-based target-enrichment strategy for NGS. Genomic DNA was sonicated and dispersed in oil into picoliter-sized aqueous droplets, which were merged with a custom dropletized target enrichment primer library. The resulting microdroplet emulsion was subjected to PCR amplification. Emulsion PCR products were purified and subjected to secondary PCR to incorporate NGS sequencing adaptors and indexes for individual sample tracking. Indexed samples from 96 individuals were pooled and loaded onto massively parallel next-generation sequencers for 2 × 150 base paired-end reads.

### NGS dosage analysis

Quantitative dosage analysis of NGS data was performed to determine copy number abnormalities indicative of deletions or duplications in exon and promoter regions. The analytic accuracy and reproducibility of NGS dosage analysis using in-house-developed review software was characterized previously [9] and approved by the New York State Department of Health prior to clinical use. Pseudogene reads were circumvented through primer design and alignment filters for NGS data analysis. For *PMS2* exon 9, exons 11–15, and their flanking regions, this approach was supplemented by dosage quantification involving previously defined paralogous sequence variants (PSVs) between *PMS2* and *PMS2CL*, its highly homologous pseudogene. Approximately 2000 NGS amplicons were used to interrogate coding exons and limited flanking intron regions of tested genes.

### Confirmatory LR analysis

The LRs discussed herein affected regions ranging from a few hundred bases to several kilobases. All samples positive for a putative LR on NGS were confirmed using procedures previously validated with positive and negative controls [9]. Most often, LRs were confirmed through targeted microarray-CGH and/or MLPA analysis. In some instances, LR findings on NGS were confirmed solely by a second confirmatory NGS result. For microarray-CGH, approximately 9600 probes interrogated coding exons, limited flanking intron regions, and promoters. Microarray probe design was optimized to avoid known pseudogene regions and included the use of flanking intronic probes in certain genes. Probe signals were analyzed using laboratory-developed software that compared the ratio of bound sample DNA to that of a differentially labeled reference DNA to identify regions of altered copy number. The amplitude of probe clusters was analyzed to elucidate the nature of the LR. MLPA was run using the SALSA MLPA gene-specific kits according to manufacturer specifications (MRC Holland. Amsterdam, Netherlands).

A targeted PCR assay was used to confirm LRs detected initially by NGS. Targeted PCR uses primer pairs that span a specific region involved in the putative LR, generally a breakpoint. The assay used long-range PCR amplification conditions when needed, depending on the size of the affected region. Mutation-specific PCR products were amplified, visualized using gel electrophoresis, and further characterized by downstream Sanger sequencing analysis. In addition to analyzing relative copy number to detect deletions and duplications, the NGS assay included custom amplicons designed to detect a previously characterized inversion of *MSH2* exons 1–7 [10]. The inversion was detected on NGS using mutation-specific components that did not amplify the inversion directly but served as a screening tool to trigger additional confirmatory work via targeted PCR.

For the *PMS2* gene, part of which is highly homologous to the *PMS2CL* pseudogene, putative LRs in exons outside the pseudogene region were tested using NGS dosage analysis and confirmed using MLPA. However, putative deletions or duplications that were contained entirely within exon 9 or exons 11–15 were confirmed using *PMS2*- and *PMS2CL*-specific sequencing analysis and/or locus-specific, long-range PCR.

**Fig. 1:**
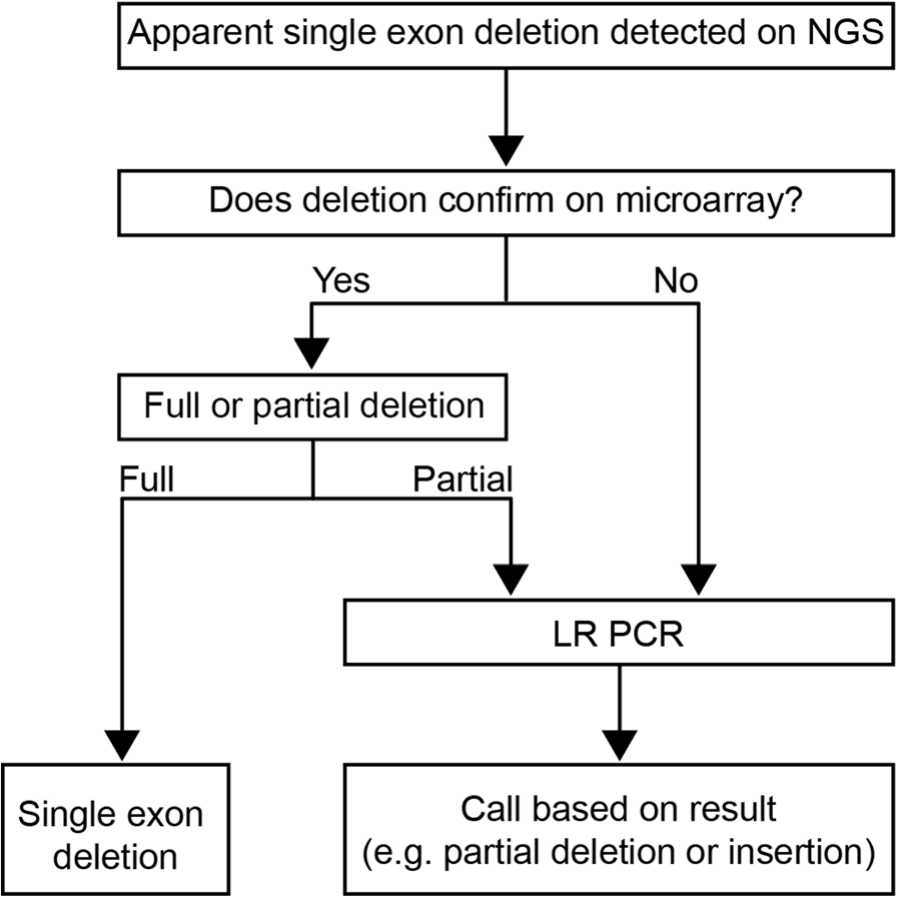
Schematic of follow-up testing to confirm type of large rearrangement (LR) following initial detection via next-generation sequencing (NGS)

Putative single-exon deletions required additional scrutiny, as true single-exon deletions and artifactual single-exon deletions can look the same on amplification-based NGS. The presence of a heterozygous variant under a primer can cause the artifactual appearance of a deletion due to PCR allele drop-out. Therefore, the sequence under the primers for the affected exon was screened for the presence of any heterozygous sequence variants. Retroelement insertions (RE) were detected by the NGS assay when they manifested through a reduced amplicon copy number. If the individual carried no heterozygous variants in the relevant region, additional studies were employed to distinguish between a possible deletion and an RE insertion, as detailed in Fig. 1. A confirmatory assay, such as targeted microarray-CGH, typically was used next. If microarray-CGH revealed a full exon deletion, the result was reported as such. Cases in which the exon appeared partially deleted on microarray-CGH were characterized further using long-range PCR and Sanger sequencing of the product to identify breakpoints. If microarray-CGH results were discordant with the initial NGS LR results (i.e. negative), long-range PCR and Sanger sequencing were performed to determine whether the LR was actually an RE insertion, which is often not detected by microarray-CGH.

### Assessment of pathogenicity

Long-range PCR and sequencing of the mutant product was used to define deletion breakpoints or RE insertion points for the purpose of variant classification. Additional investigation was conducted to characterize an LR precisely in every case where variant classification could be impacted. In general, LRs were classified as deleterious mutations (DM) or suspected deleterious mutations (SDM) and thus considered pathogenic based on disruption or loss of critical gene regions, or consensus splice junction removal that is likely to produce abnormal RNA splicing. For instance, RE insertions into critical domains within coding regions were considered pathogenic. Alternatively, in-frame deletions or REs that insert within a non-critical region or a region of unknown function were classified as variants of uncertain significance (VUS).

## Results

A comprehensive strategy that employed NGS followed by confirmatory analyses detected 3461 LRs among 376,159 tested individuals (Table 1). LRs were found in all genes for which LR analysis was performed. The largest proportion of pathogenic LRs were identified in *BRCA1* (27.4%), followed by *PMS2* (11.7%)*, CHEK2* (11.1%) and *MSH2* (8.9%) (Table 2). The high number of pathogenic LRs detected in *PMS2* is of particular interest, as 34.5% of LRs in this gene occurred within exon 9 or exons 11–15. These exons share significant sequence homology with the *PMS2CL* pseudogene, which may confound result analysis and interpretation. This finding highlights the importance of additional confirmatory work to establish that any putative LR in this region lies within *PMS2* (which is clinically significant) and not in *PMS2CL* (which is not clinically significant).

**Table 1:** Distribution of Pathogenic and Variant of Uncertain Significance (VUS) Large Rearrangements (LRs)^a^

Frequency	Deletion	Duplication	Insertion	Inversion	Triplication	Total
VUS	96 (4.5%)	997 (79.1%)	12 (22.2%)	0	13 (54.2%)	1118 (32.3%)
Pathogenic	2017 (95.5%)	264 (20.9%)	42 (77.8%)	8 (100%)	11 (45.8%)	2342 (67.7%)
Total	2113	1261	54	8	24	3460

^a^One complex *RAD51C* LR, composed of both a multi-exon deletion and a multi-exon duplication, is not included

**Table 2:** Distribution of Pathogenic Large Rearrangements (LRs) by gene^a^

Frequency	Deletion	Duplication	Insertion	Inversion	Triplication	Total
*APC*	31	2	0	0	0	33 (1.4%)
*ATM*	104	5	28	0	0	137 (5.8%)
*BARD1*	45	9	0	0	0	54 (2.3%)
*BMPR1A*	7	0	0	0	0	7 (0.3%)
*BRCA1*	479	162	0	0	0	641 (27.4%)
*BRCA2*	82	7	11	0	10	110 (4.7%)
*BRIP1*	44	4	0	0	0	48 (2.0%)
*CDH1*	19	2	0	0	0	21 (0.9%)
*CDKN2A* p14ARF	2	0	0	0	0	2 (0.1%)
*CDKN2A* p16/p14ARF	3	0	0	0	0	3 (0.1%)
*CHEK2*	256	3	0	0	0	259 (11.1%)
*EPCAM*	17	0	0	0	0	17 (0.7%)
*GREM1*	0	2	0	0	0	2 (0.1%)
*MLH1*	61	20	1	0	0	82 (3.5%)
*MSH2*	192	9	0	8	0	209 (8.9%)
*MSH2/EPCAM*	26	0	0	0	0	26 (1.1%)
*MSH6*	26	0	0	0	0	26 (1.1%)
*MYH*	10	0	0	0	0	10 (0.4%)
*NBN*	46	0	0	0	0	46 (2.0%)
*PALB2*	146	17	1	0	0	164 (7.0%)
*PMS2*	260	14	1	0	0	275 (11.7%)
*PTEN*	4	1	0	0	0	5 (0.2%)
*RAD51C*	99	6	0	0	1	106 (4.5%)
*RAD51D*	24	0	0	0	0	24 (1.0%)
*SMAD4*	1	0	0	0	0	1 (<0.1%)
*STK11*	16	1	0	0	0	17 (0.7%)
*TP53*	17	0	0	0	0	17 (0.7%)
Total	2017	264	42	8	11	2342

^a^One complex *RAD51C* LR, composed of both a multi-exon deletion and a multi-exon duplication, is not included

**Fig. 2:**
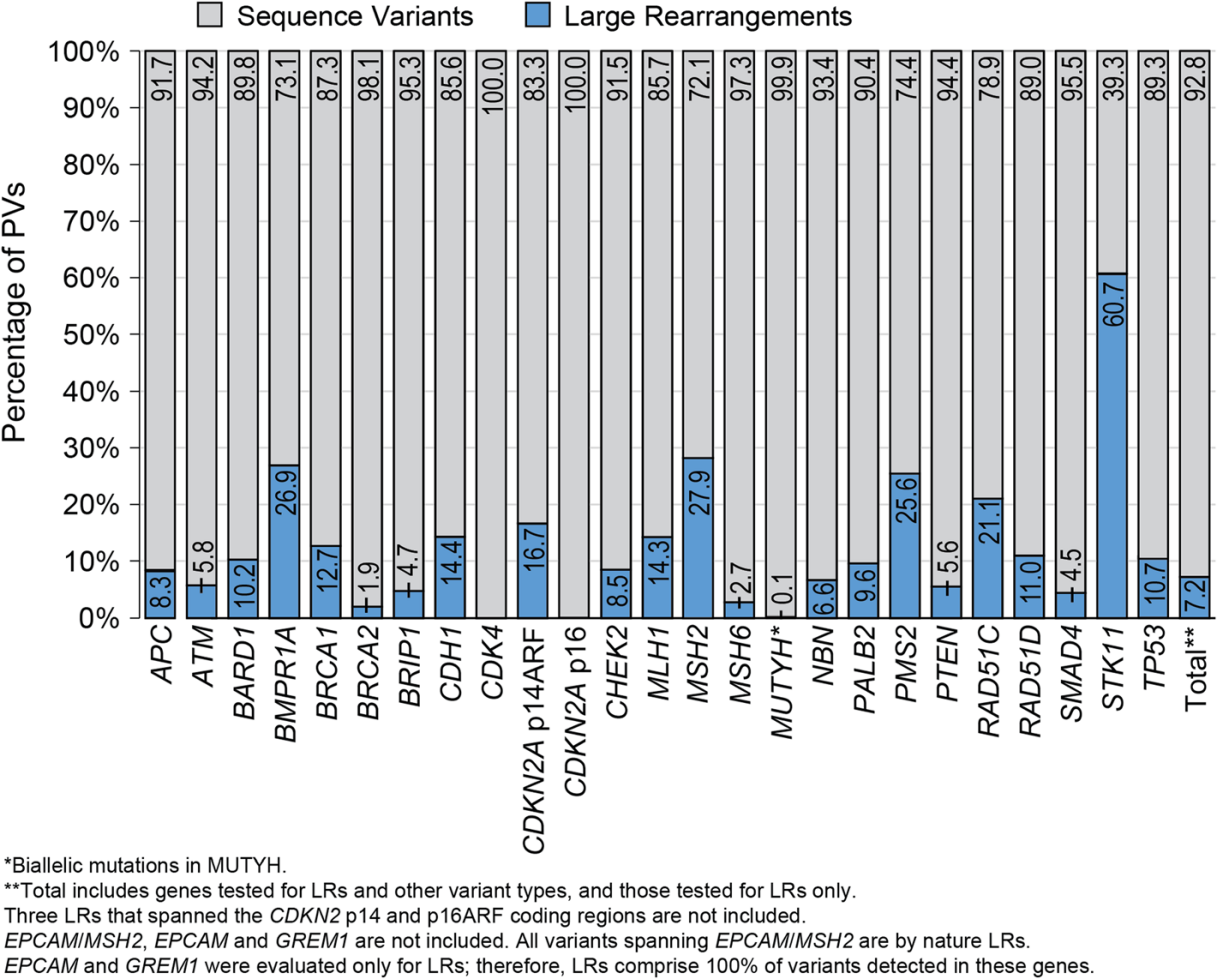
Proportion of pathogenic sequence variants and large rearrangements (LRs) identified by gene

Overall, LRs accounted for 7.2% of all pathogenic variants detected across all 28 panel genes (Fig. 2). However, the prevalence of LRs varied by gene. LRs were most prevalent in *STK11* (60.7% of all pathogenic variants identified in the gene), *MSH2* (27.9%), *PMS2* (25.6%), *BMPR1A* (26.9%), *RAD51C* (21.1%), and *CDKN2A* p14ARF (16.7%) (Fig. 2). Of the 2336 individuals found to carry pathogenic LRs, 164 (7.0%) had at least one other germline PV. Among these, 157 individuals had sequence variants, and seven carried an additional LR in a separate, non-contiguous gene.

Nearly all (95.5%) of deletions included here were pathogenic (Table 1). with the remaining 4.5% being classified as VUS (Table 1). Only 4.5% of deletions were classified as VUS (Table 1), including those found in *GREM1* and *CDK4*, where no pathogenic deletions were observed. Deletions represented 86.1% of pathogenic LRs detected and were seen in almost all tested genes (Table 2).

**Fig. 3:**
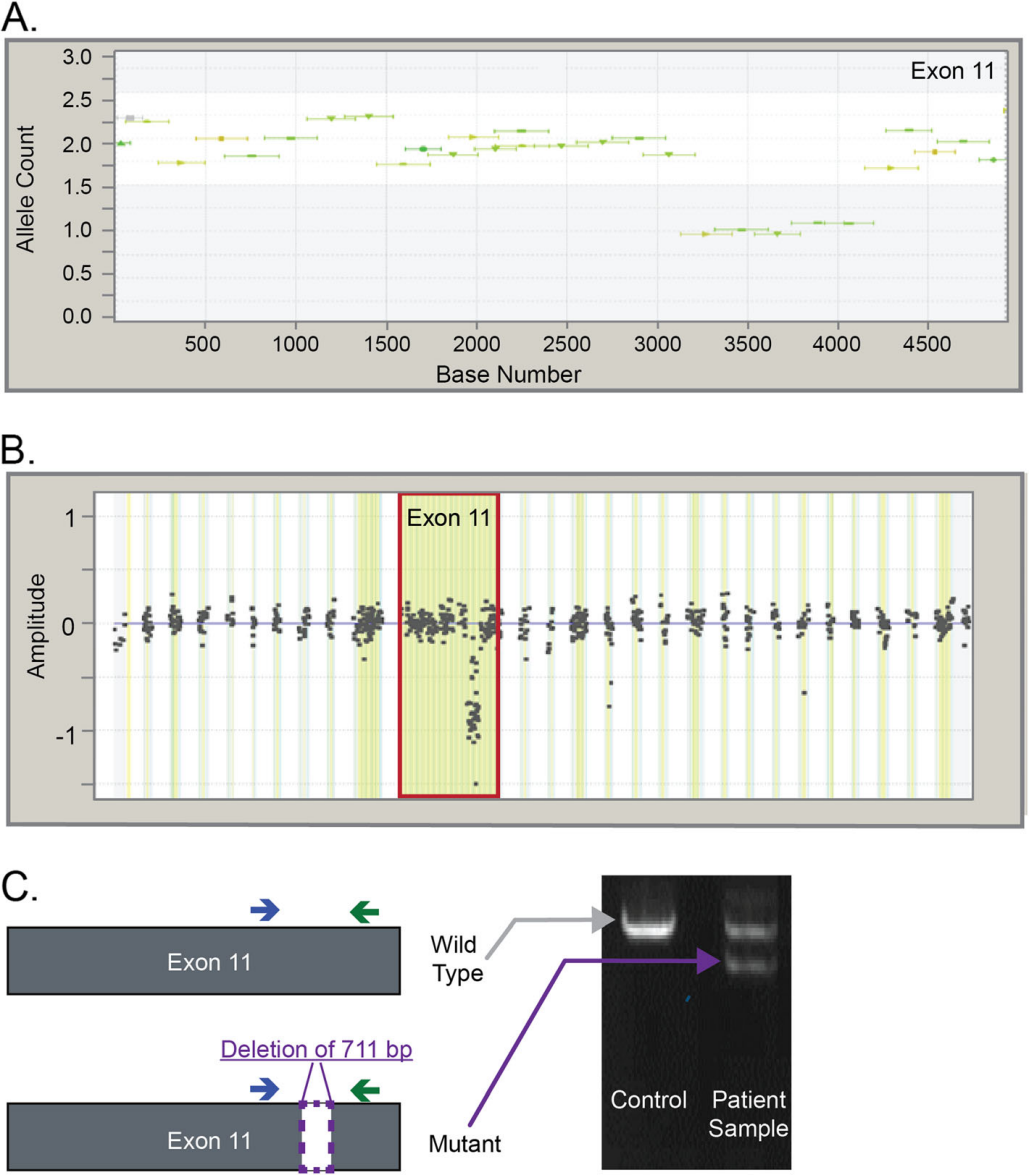
Laboratory data indicating the presence of a partial deletion in *BRCA2.* **a** Next-generation sequencing (NGS) data showing a partial deletion in exon 11 of *BRCA2*. Exons at normal dosage align to 2 alleles on the y-axis, whereas in deletions they align to one allele. **b** Microarray-CGH data showing a partial deletion. Microarray probe clusters at normal dosage center at 0 on the y-axis, whereas deletions center between approximately − 0.75 and − 1.0. **c** Schematic and results of targeted PCR visualized using gel electrophoresis. The sample contains a mutant PCR product of smaller size. Deletion breakpoints were determined by Sanger sequencing (data not shown)

A total of 41 unique pathogenic and VUS partial deletions were detected here, present in 79 individuals. Figure 3 illustrates one partial deletion in *BRCA2*. NGS initially showed that amplicons covering a sub-region of exon 11 (spanning ~ 1000 bp in size) were present in only one copy (Fig. 3a). This partial deletion was also investigated by targeted microarray-CGH, as directed by the process flow detailed in Fig. 1, and a decreased relative amplitude of probe clusters in this region confirmed the NGS finding (Fig. 3b). MLPA did not detect this LR because the MLPA probe binding sites for this exon did not cover the region deleted in this individual (data not shown). Targeted PCR was employed to characterize precise breakpoints and evaluate the pathogenicity of the partial deletion. The analysis revealed an in-frame deletion of 711 bp within a non-critical region. As a result, this LR was classified as VUS.

**Fig. 4:**
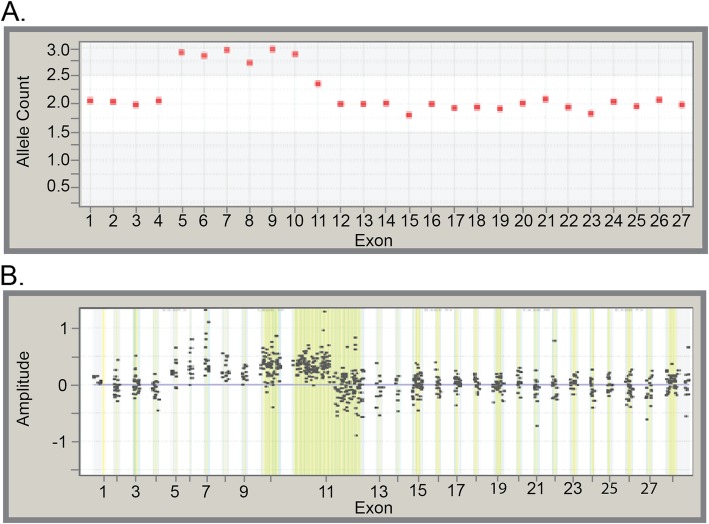
Laboratory evidence suggesting a duplication*.* **a** Next-generation sequencingNGS data showing an apparent duplication of exons 5–10 of *BRCA2*. Exons at normal dosage align to two alleles on the y-axis, whereas in duplications they align to three alleles. **b** Microarray-based comparative genomic hybridization data clearly demonstrate that the duplication also includes the first half of exon 11. Microarray probe clusters at normal dosage center at zero on the y-axis, whereas duplications center to approximately 0.4–0.5

Duplications were detected in each of the 26 genes evaluated for LRs; however, pathogenic duplications were identified in only 16 genes (Table 2). Overall, duplications accounted for 11.3% of all detected pathogenic LRs, and 79.1% of all duplications were classified as VUS (Table 1). Five unique partial duplications (pathogenic variants and VUS) were detected in seven individuals. One partial duplication in *BRCA2* is depicted in Fig. 4. NGS showed that exons 5–10 were elevated to allele counts of approximately 3, which is consistent with duplication (Fig. 4a). Confirmatory microarray-CGH refined this duplication further and demonstrated that a portion of exon 11 was included in the duplication as well (Fig. 4b). Follow-up targeted PCR established that the duplicated segment occurred in tandem with the native gene, in a head-to-tail orientation (Fig. 4c). Such a configuration is predicted to result in abnormal protein production and/or function, and the variant was classified as DM.

**Fig. 5:**
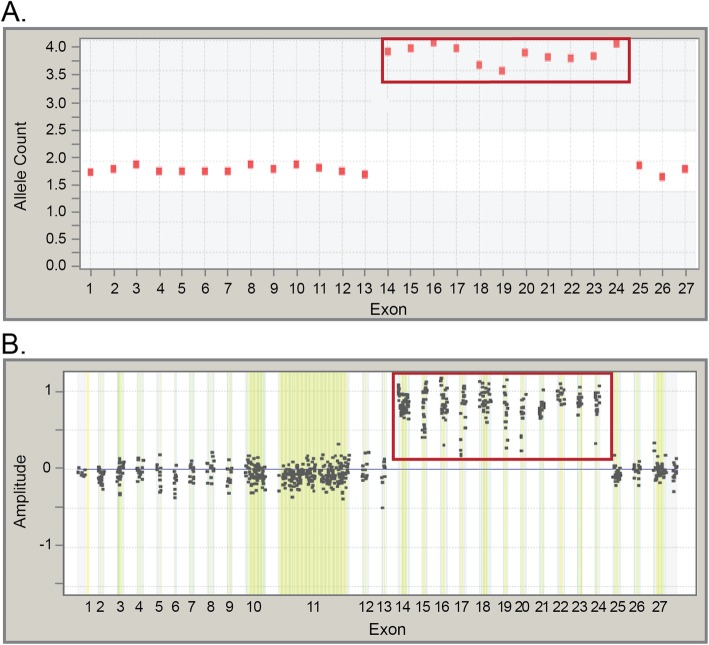
Laboratory evidence of a triplication found in *BRCA2.* **a** Next-generation sequencingNGS data showing a triplication of exons 14–24 of *BRCA2*. Exons at a normal dosage align to 2 alleles on the y-axis, whereas triplicated exons align to about four alleles. **b** Microarray-based comparative genomic hybridization data showing a triplication of exons 14–24. Microarray probe clusters at normal dosage center at zero on the y-axis, whereas triplications center at approximately 0.75–1.0

Triplications were detected in *BRCA2*, *RAD51C* and eight additional genes; however, pathogenic triplications were detected only in *BRCA2* and *RAD51C* (Table 2). Overall, triplications accounted for 0.5% of all pathogenic LRs, and 54.2% of triplications were classified as VUS (Table 1). One unique pathogenic *BRCA2* triplication was detected in this study (Fig. 5). This triplication has been identified in multiple individuals, most traced to a large North American kindred. NGS amplicons in exons 14–24 were elevated to allele counts of approximately four, indicating the presence of two additional copies of this region of the gene (Fig. 5a). Targeted microarray-CGH was consistent with the presence of a triplication, with probe clusters centered at an amplitude of about 0.75 for exons 14–24 of *BRCA2* (Fig. 5b). Additional analysis by long-range PCR confirmed that at least two of the three copies of exons 14–24 were present in tandem at the native locus, in a head-to-tail orientation. This LR is in-frame but involves most of the DNA binding domain for *BRCA2*, and it therefore was classified as SDM.

Forty-two pathogenic RE insertions were detected in five tested genes, accounting for 1.8% of pathogenic LRs (Table 2). Only 22.2% of all RE insertions were classified as VUS (Table 1). Finally, pathogenic inversion of *MSH2* exons 1–7 [10] was seen in eight individuals, accounting for 0.34% of all pathogenic LRs found in the cohort (Table 2).

## Discussion

As clinical use of genetic testing for hereditary cancer risk has become more prevalent, professional societies have issued gene-specific medical management recommendations based on pathogenic variants identified in validated cancer-risk genes [11, 12]. Compared with sequence mutations, however, the contribution of LRs in disease risk assessment has been underappreciated.

This analysis of data collected over a 3.5-year period found that LRs accounted for more than 7% of pathogenic variants identified among individuals tested for a clinical suspicion of hereditary cancer risk. LRs were much more common in certain genes, representing a substantial proportion of clinically actionable findings. For example, pathogenic variants in *STK11* are diagnostic of Peutz-Jeghers syndrome, which is associated with a high risk of breast, colorectal, endometrial, pancreatic, gastric, ovarian, and other cancers [12]. As such, individuals with pathogenic *STK11* variants are recommended to receive increased cancer screening at a younger age [12]. The analysis found that LRs accounted for more than 60% of pathogenic *STK11* variants identified. In addition, LRs accounted for more than 20% of pathogenic variants in *BMPR1A*, *CDKN2A* p14ARF, *MSH2/EPCAM*, *PMS2*, and *RAD51C*. The substantial proportion of LRs seen in cancer predisposition genes highlights the need to identify and classify this type of variant accurately for appropriate medical management.

Various types of LRs that are difficult to detect and/or characterize include partial deletions or duplications, inversions and RE insertions. In addition, genes bearing significant sequence homology to pseudogenes must also be handled with care to ensure that only pathogenic variants present in the native gene are reported. This is particularly relevant for *PMS2* where 34.5% of pathogenic LRs in *PMS2* occurred in the pseudogene region (i.e., involving any combination of exons 11–15). Specific LRs, such as the *MSH2* inversion of exons 1–7, may require design of breakpoint-specific elements for detection. LRs resulting in reduced dosage of the affected regions in our NGS assay, including but not limited to RE insertions, require additional scrutiny. In this analysis, RE insertions were detected in 42 individuals. Furthermore, 63 individuals from this study carried a partial exon deletion, two carried a partial exon duplication, eight carried the *MSH2* inversion, and 95 carried a pathogenic LR within the *PMS2* pseudogene region. Without the comprehensive detection and confirmation strategy employed here, 9% of LRs identified in this study might have been missed, potentially leading to inappropriate medical management in as many as 210 individuals referred for hereditary cancer testing.

Hereditary pan-cancer panel tests use NGS technology to deliver simultaneous, multi-gene analyses with reliable sequence variant detection. This work has demonstrated that NGS dosage analysis also accurately detects the presence of many LR types, including commonly observed deletions and duplications as well as less common insertions, triplications, and partial deletions/duplications.

This analysis also demonstrates the importance of multiple assays for LR identification and characterization. For instance, a partial deletion of *BRCA2* exon 11 was identified here by NGS and microarray-CGH but was missed by MLPA due to limitations in coverage. Although MLPA historically has been used as a primary LR identification method, it is clear that the lack of complete coverage may lead to missed diagnostic opportunities if the method is used in isolation. This is especially true of small LRs or RE insertions that are less likely to overlap with MLPA probe binding sites. Long-range PCR and Sanger sequencing analyses were performed whenever possible to establish the LR breakpoints and evaluate pathogenicity.

A strength of this study is that it represents a large consecutive cohort of individuals tested based on clinical suspicion of hereditary cancer risk. In addition, the study employed multiple confirmatory testing methods to detect variants, allowing comprehensive evaluation of the quantity and types of variants identified as part of hereditary cancer testing. NGS dosage analysis detects unbalanced LRs such as deletions and duplications involving one or more exons. One limitation of the study is that analyses of *GREM1* and of *MSH2* inversion elements were not included on the panel for the full time period included in this analysis. As such, variants in these genes might be underrepresented in the dataset. Also, as is true for all gene panels, this assay does not detect every possible type of genomic rearrangement. The NGS assay employed here includes custom regions designed to interrogate for specific complex LRs (e.g., the 10-Mb inversion mutation involving *MSH2* exons 1–7). However, additional complex LRs, such as RE insertions, were detected initially through reduced dosage of affected regions in the NGS assay. These were further characterized with additional analyses that aided in the interpretation of their clinical significance.

## Conclusions

This work shows that NGS dosage analysis can accurately detect the presence of various types of genomic LRs as part of a comprehensive genetic testing strategy. LRs account for a significant proportion of pathogenic variants identified in 28 cancer risk genes tested in our laboratory. Some pathogenic LRs are technically challenging and otherwise might be missed by routine genetic testing. In the absence of this comprehensive testing strategy, 9% of LRs identified in this testing population might have been missed, potentially leading to inappropriate medical management in as many as 210 individuals referred for hereditary cancer testing. These results highlight that a robust and accurate LR-identification strategy is an essential component of a high quality genetic testing program, enabling clinicians to optimize patient medical management decisions for patients and their family members at risk of hereditary cancer.

## Data Availability

The datasets analyzed during the current study are not publicly available due to patient privacy.

## Abbreviations

DM: Deleterious mutation
LR: Large rearrangement
microarray-GCH: microarray-based comparative genomic hybridization
MLPA: multiplex ligation-dependent probe amplification
NGS: Next-Generation Sequencing
PSV: paralogous sequence variant
RE: retroelement
SDM: Suspected Deleterious Mutation
VUS: Variant of Uncertain Significance
